# Extended Focused Assessment with Sonography for Trauma in the Emergency Department: A Comprehensive Review

**DOI:** 10.3390/jcm14103457

**Published:** 2025-05-15

**Authors:** Federico M. Bella, Alessandra Bonfichi, Ciro Esposito, Christian Zanza, Abdelouahab Bellou, Domenico Sfondrini, Antonio Voza, Andrea Piccioni, Antonio Di Sabatino, Gabriele Savioli

**Affiliations:** 1 Department of Internal Medicina, IRCCS Fondazione Policlinico San Matteo, 27100 Pavia, Italy; federicomaria.bella01@universitadipavia.it (F.M.B.); alessandra.bonfichi@gmail.com (A.B.); a.disabatino@smatteo.pv.it (A.D.S.); 2 Unit of Nephrology and Dialysis, ICS Maugeri, University of Pavia, 27100 Pavia, Italy; ciro.esposito@unipv.it; 3 Geriatric Medicine Residency Program, University of Rome “Tor Vergata”, 00133 Rome, Italy; christian.zanza@gmail.com; 4 Institute of Sciences in Emergency Medicine, Department of Emergency Medicine, Guangdong Provincial People’s Hospital (Guangdong Academy of Medical Sciences), Southern Medical University, Guangzhou 510080, China; abellou402@gmail.com; 5 Maxillofacial Unit, Emergency Department, IRCCS Fondazione Policlinico San Matteo, 27100 Pavia, Italy; d.sfondrini@smatteo.pv.it; 6 Department of Biomedical Sciences, Humanitas University, Pieve Emanuele, 20072 Milan, Italy; antonio.voza@humanitas.it; 7 Department of Emergency Medicine, Fondazione Policlinico Universitario A. Gemelli IRCCS, 00168 Rome, Italy; andrea.piccioni@policlinicogemelli.it; 8 Department of Internal Medicine and Medical Therapeutics, University of Pavia, 27100 Pavia, Italy; 9 Emergency Medicine and Surgery, IRCCS Fondazione Policlinico San Matteo, 27100 Pavia, Italy

**Keywords:** eFAST, FAST, trauma, sonography, ABCDE, echocardiography, emergency medicine, intra-abdominal fluid, critical care, resuscitation, pneumothorax, pleural effusion, cardiac tamponade, blunt abdominal trauma, intraperitoneal, management, ultrasound, emergency department

## Abstract

The Extended Focused Assessment with Sonography for Trauma (eFAST) plays a crucial role in the emergency department (ED) by providing rapid and non-invasive diagnostic information in trauma patients. It is a diagnostic-free fluid detection technique that uses sonography to assess trauma in different anatomical windows of the chest and abdomen and has been accepted in multiple studies as the initial diagnostic tool for torso injuries in blunt abdominal trauma. By promptly identifying potentially life-threatening injuries, such as haemoperitoneum, haemothorax, and cardiac tamponade, eFAST facilitates timely intervention and improves patient outcomes in the ED. The eFAST exam is reliable, with high sensitivity and specificity, even when performed by non-radiological personnel, saving time and resources in the chaotic emergency environment. This review aims to assess the diagnostic reliability and limitations of eFAST in different trauma conditions and to outline its goals in trauma critical care and in “ABCDE” resuscitation.

## 1. Introduction

Ultrasound (US) is a cheap, rapid, and non-invasive technique for the evaluation of trauma patients with abdominal and thoracic trauma, with the best performance in the detection of air and free fluid.

US has been used in trauma evaluation in Europe since the 1970s, but it was not adopted in North America until the 1990s, when the acronym FAST was introduced as “Focused Abdominal Sonography for Trauma [1]” in 1996. As the aim of the FAST exam was expanded to include broader trauma assessment, the acronym was changed to “Focused Assessment with Sonography for Trauma” [2]. In 2003, the American College of Emergency Physicians published a technical guide called “The FAST Exam”, and then the American College of Surgeons Committee on Trauma included FAST in the Advanced Trauma and Life Support (ATLS) guidelines. Finally, in 2004, the FAST exam was further implemented to include an extended view of the chest to look for pneumothorax (PTX) and pleural effusion, creating the acronym eFAST [3,4].

The use of US in emergency care has improved its quality, particularly in terms of the procedural safety, timeliness of care, diagnostic accuracy, and cost reduction [5]. Prior to its development, more invasive procedures, including surgery, were required.

The progressive incorporation of US into routine trauma care had led to a reduction in diagnostic peritoneal lavage (DPL) and may lead to a decrease in the use of computed tomography (CT), resulting in a significant reduction in the cost of care for trauma patients [6] and a reduction in radiation exposure for ED patients. Moreover, US has considerable advantages, including bedside availability for unstable patients who cannot be transferred for CT, the identification of abdominal bleeding and timely surgical intervention, its ease of use, and reproducibility [1].

In addition, US assists with intubation by visualising the airway, confirming correct tube placement, and detecting PTX.

Incorporating US training into emergency physician education and using it for point-of-care medicine might significantly improve performance and inpatient outcomes by shifting the diagnostic approach from a systematic, organ-based assessment to a multiple-target, disease- and problem-based approach [7].

## 2. Materials and Methods

**Figure 1:**
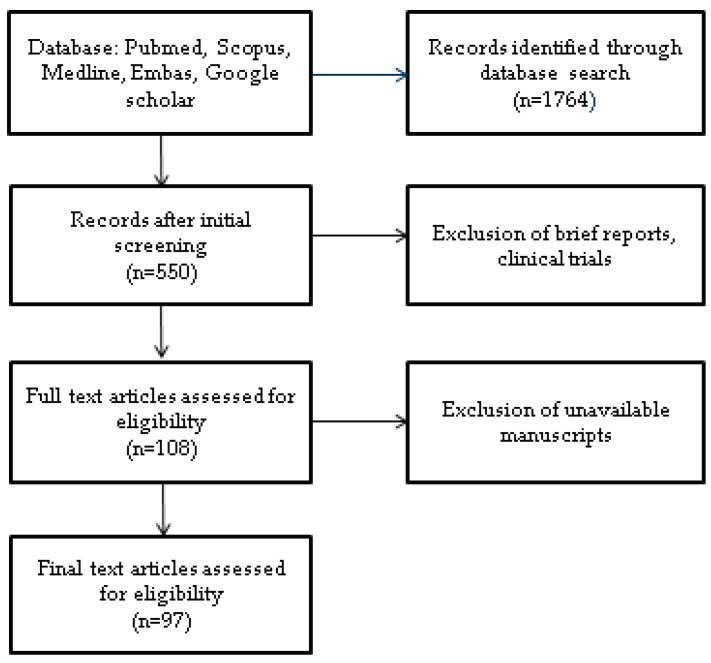
Materials and methods.

For the timeline from January 1984 to January 2024, a search was conducted on the main scientific platforms: Pubmed, Scopus, Medline, Embase, and Google scholar. Using the MeSH database, we initially found a total of 1764 articles, matching “eFAST, ultrasound AND emergency department, emergency medicine, trauma management, ABCDE”. A second screening reduced the number of eligible articles to a total of 550, from which abstracts of meetings, unavailable manuscripts (original articles, meta-analyses, and systematic reviews), original articles without abstracts, and brief reports were excluded, leaving only relevant articles related to emergency medicine, critical or intensive care medicine, and acute medicine. In addition, the reference lists of each article were reviewed to find relevant articles to include, and finally, 97 articles were analysed in this clinical review. (Figure 1).

## 3. Results

### 3.1. eFAST Execution Technique

eFAST is a bedside test that can be performed directly by the emergency physician. It is performed with the patient in the supine position. However, one study suggested that the adoption of a Trendelenburg position may be more appropriate for detecting free fluid in the hepatorenal, hepatosplenic, and pleural spaces, while the reverse Trendelenburg should be used for pelvic evaluation and haemothorax assessment, especially in the presence of small amounts of fluid [8].

When performing the examination, a multipurpose curvilinear probe operating at low frequencies (3–5 GHz) is the best choice, providing the best versatility in different anatomical windows, with deep imaging penetration and an overall fast exam [9]. A phased array transducer may not provide ideal cardiac imaging, but the eFAST exam is not intended to replace a thorough echocardiography [10] and, therefore, justifies the limitation mentioned.

According to Brun et al. a properly trained practitioner should be able to perform it in an average time of 3.5 min in the field, or an average of 3.9 min during patient transfer [11].

When performing US, a systematic approach to the exam is recommended for optimal information collection. This includes asking simple questions with binary yes-or-no answers:

- Is there any free fluid or air? (To assess post-traumatic fluid effusion in the peritoneum and pericardium.).
- Are cardiac functions normal? (To assess for possible hypotension or tachycardia.).
- Is there any lung sliding present? (To rule out pleural effusion.).

The FAST exam can effectively detect free fluid with limited scan planes, prioritizing fluid over organ detail. Its effectiveness depends on the minimum amount of free fluid or air in different anatomical windows [12]:

- In the abdominal region, free fluid is visible in the hepatorenal or splenorenal spaces if there is >500 mL, on average. A study by Branney et al. estimated the minimum detectable free-fluid volume in 100 patients to be 619 mL [13]. Abrams et al. concluded that the FAST exam is more accurate in detecting small amounts of abdominal fluid in the Trendelenburg position than in the supine position (minimum detectable free fluid > 400 mL) [8].
- In the pelvic region, according to Jehle et al., the average minimum detectable free fluid is 157 mL [14]. Physiological pelvic fluid is limited to 50 mL in the Douglas pouch in fertile women. Volumes greater than 50 mL suggest pathology, most likely due to trauma.
- When performing the extended-view US of the lungs (LU), the minimum volume of free fluid required to detect a pleural effusion is as low as 20 mL [15].

**Figure 2:**
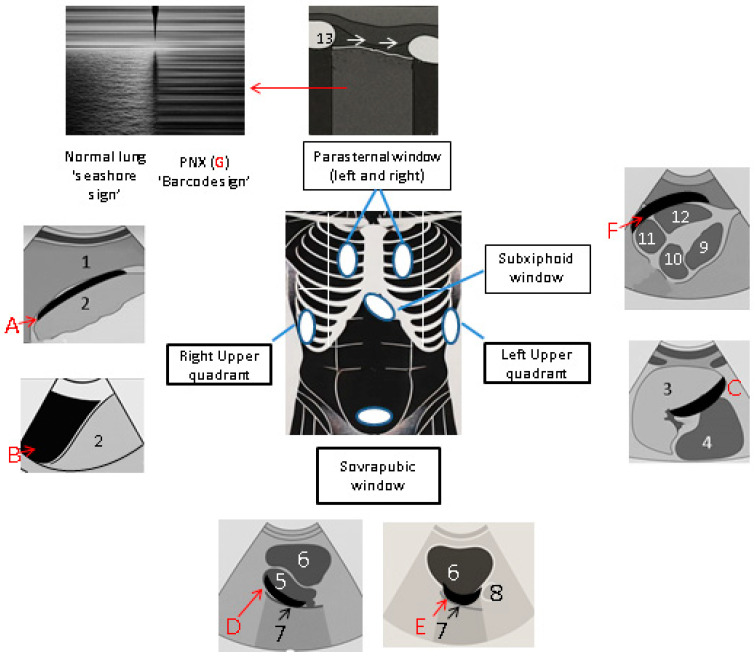
By placing the probe in the right anterior axillary position, it is possible to assess the following: a haematic effusion in Morrison’s pouch (**A**), located between the liver (1) and the kidney (2); or a pleural effusion (**B**) accumulating in the costodiaphragmatic recess (e.g., haemothorax). By placing the probe along the left posterior axillary line, it is possible to assess the presence of a left-sided haemothorax above the diaphragm in the costophrenic angle, or a haemorrhage between the spleen (3) and the left kidney (4) in the perisplenic region (**C**). Abdominal fluid accumulates in the Douglas pouch (**D**) between the uterus (5) and the rectum (7) in women and in the rectovesical space in males (**E**). Bladder (6). Prostate (8). With the probe placed in the transverse epigastric position, the pericardial effusion appears as a rim of fluid surrounding the ventricles (**F**). Left ventricle (9), left atrium (10), right atrium (11), right ventricle (12). The heart is seen in the long-axis view. Extended FAST allows the assessment of the presence of a pneumothorax (**G**) in a trauma patient by placing the probe sagittally in all four quadrants of each hemithorax in the parasternal view. In normal lung parenchyma, respiratory movements cause variations in the reflected echoes. When the lung is fully expanded, the “seashore sign” can be observed in M-mode. A physiological sliding of the lung from left to right can be seen in B-mode images. If the lung is not fully expanded, the “barcode sign” appears. The total reflection of echoes at the pleural line is caused by air between the two pleural layers. Rib (13).

Emergency sonographic techniques include focused echocardiography, as well as thoracic and abdominal US [16]. The most widely used technical approach consists of the evaluation of four sonographic windows [6], and up to 6 in the extended version, as it also includes the parasternal views (P1 and P2) to assess the thorax [17]. These should be examined in a circular manner, approaching the patient from the right side, looking for the presence of fluid or air presence or organ damage (Figure 2):

1. The subxiphoid/subcostal space is examined to assess the condition of the pericardium, looking for signs of pericardial effusion, pericardial tamponade, wall motion abnormalities, and the adequacy of right ventricular filling. The probe should be positioned in the subxiphoid fossa, directed towards the patient’s head, and advanced towards the left midclavicular line and left shoulder. The US image should outline all four chambers of the heart, with the right ventricle being in contact with the upper border of the liver, which is also one of the most common sites of fluid collection. US signs of pericardial tamponade include the diastolic collapse of the right ventricle and atrium, exaggerated respiratory variations in AV Doppler inflow velocities, and IVC plethora. Correctly differentiating pathological diastolic collapse from physiological systolic collapse can be difficult in the emergency setting without an ECG. The use of the M-mode Doppler helps to assess diastolic displacement and blood velocity changes [18].
2. The perihepatic view is examined to assess the Morrison’s pouch, looking for fluid. Within the abdominal cavity, fluid tends to collect in the lateral recesses, due to an anterior bulging of the peritoneum caused by the spine. The superficial observational window starts from the inferior costal margin on the anterior abdomen in the coronal plane, down to the mid-posterior axillary line. The sonographic image should outline the inferior border of the liver, the inferior pole of the kidney, and the right costodiaphragmatic recess of the pleural space [19,20].
3. The hypogastric region is examined using the suprapubic window to examine the pelvis with transverse, then longitudinal, scans. The superficial observational window extends from the pubic bone upwards towards the umbilicus. The superficial window is from the pubic bone to the navel. In men, fluid may accumulate in the rectovesical pouch. In females, it typically accumulates in the Douglas pouch, anterior to the uterus. It should be noted that females with a posteriorly flexed uterus are more likely to have fluid collection on the superior border of the bladder, due to uterine displacement [21].
4. The left upper quadrant (LUQ) is examined to assess the splenorenal, perisplenic, and left paracolic spaces in search of fluid. The superficial window goes from the left posterior axillary line to the left mid-axillary line in the coronal plane. The sonographic image should outline the inferior and superior poles of the spleen, the inferior pole of the kidney, and the left costodiaphragmatic recess of the pleural space [22].
5. In the extended FAST view, the pleural space is assessed for pleural effusion or PTX. The optimal sonographic window is a bilateral longitudinal scan along the midclavicular line over the right and left hemithorax (P1 and P2 parasternal views). The “bat sign” helps to identify the pleural line between the ribs, and the presence of lung sliding rules out PTX [23].

The “lung point”, where the visceral pleura separates from the parietal pleura due to the presence of air in the pleural space, indicates PTX [24]. In a healthy lung, the US beam captures the movement of the pleural layers, producing a characteristic “sandy beach” or “seashore” sign. Normal lung movement appears as a grainy, dynamic pattern (“seashore sign”) below the pleural line. In PTX, air in the pleural space abolishes lung sliding and produces the “stratosphere” or “barcode” sign, such as parallel static lines. The pleural space and costodiaphragmatic recess show curvilinear echogenic lines with inspiratory distortion (“curtain sign”), whereas pleural effusions or haemothorax appear anechoic or variably echogenic, depending on fluid composition [1]. When performing eFAST, clinicians must consider the changing echogenicity of blood over time. Initially, blood appears anechoic, similar to ascitic fluid, but as it coagulates, it becomes hyperechoic and uneven, collecting around the undersides of organs. Over time, as clots lyse, blood becomes anechoic again, but sedimentation creates an echogenic–anechoic interface that can resemble soft tissue and make diagnosis difficult [25,26].

### 3.2. Clinical Application of eFAST in the ED

eFAST is indicated in trauma patients presenting to the ED for the rapid assessment of potential intra-abdominal and intrathoracic injuries. It is a valuable examination in an unstable critically ill patient, aiding ED physicians in making urgent management decisions. Although CT offers superior accuracy in the diagnosis of blunt and penetrating trauma injuries, the eFAST exam is beneficial in scenarios where time constraints make CT impractical. Furthermore, Neal et al. showed that obtaining a CT scan in this population may lead to increased mortality [27]. Previous studies have shown that bedside US may be better than chest radiography for the rapid detection of emergency conditions, such as haemothorax or pneumothorax, in trauma patients [28]. The eFAST examination includes the assessment of the peritoneal cavity, as well as the analysis of the pericardial and pleural spaces, and is a proven and useful technique for the evaluation of bleeding after traumatic injury, particularly blunt and penetrating abdominal and/or thoracic trauma [29,30]. Especially in haemodynamically unstable patients, the FAST examination has been shown to be useful in the diagnosis of intraperitoneal bleeding from splenic or hepatic injury. One study showed that despite a high false-negative rate (approximately 49%), there was no increase in mortality or the ICU length of stay [31,32]. Historically, providers performed a diagnostic peritoneal lavage (DPL) to detect haemoperitoneum. This shift in the diagnostic approach from longer and more invasive exams, such as DPL and CT, to a rapid and less invasive technique, has been supported by increasing evidence of the statistical reliability of the FAST exam [33]. A retrospective study aimed to compare the bedside US detection of haemoperitoneum with DPL in patients with blunt abdominal trauma (BAT). As a result, the researchers concluded that bedside US is a safe, rapid, and accurate screening technique for the detection of haemoperitoneum in patients with abdominal trauma, identifying intraperitoneal bleeding in less than one minute [34,35]. eFAST is useful in identifying blunt chest trauma, such as haemothorax and cardiac tamponade. Although blunt cardiac injures are relatively rare, US cardiac exploration should be performed in all patients with significant blunt chest trauma, particularly those with hypotension [36]. In regard to penetrating chest injuries, both the surgical literature and the emergency medicine literature support the use of focused sonography. The introduction of immediate bedside ED echocardiography has been shown to reduce the time to diagnosis of penetrating cardiac injuries and to improve both survival and neurologic outcomes [37,38]. Moreover, rapid US has been shown to be useful in ED patients presenting with shock and hypotension without a clear cause. Emergency physicians can perform RUSH (Rapid Ultrasound for Shock and Hypotension) to differentiate between hypovolaemic, obstructive, cardiogenic, and distributive forms of shock [39]. Furthermore, the use of a bedside technique in the ED during the COVID-19 pandemic showed optimal results for patient management, as the sanitization of the US equipment is much less time consuming than sanitizing the entire CT room, thus minimizing the time and risks associated with transferring positive patients between different wards [40,41,42]. In the emergency setting, eFAST could better manage ED overcrowding by providing a quick and reliable exam to monitor patients that require stabilization or prolonged observation. This could help to achieve a more timely and efficient diagnosis of patients with uncertain clinical features who require further investigation, such as those with congestive heart failure relapses or abdominal pain, decreasing their length of stay in the ED [43,44,45,46].

### 3.3. eFAST Diagnostic Accuracy

The following are the reported eFAST accuracy values in a disease-specific manner:

1. BAT.

In a study by Tso et al., the authors provided a preliminary report on the use of sonography in BAT. Patients whose abdominal work-up indicated the need for DPL or CT were evaluated sonographically within the first hour of admission. In a sample of 163 BAT patients evaluated by US, 146 were true negative (TN), 11 were true positive (TP), 5 were false negative (FN), and 1 was false positive (FP), with a sensitivity of 69%, specificity of 99%, and accuracy of 96% in the diagnosis of abdominal injuries [32].

Another study by Nnamonu et al. on a sample of 57 BAT patients (46 were TP, 6 were TN, 3 were FP, and 2 were TN) concludes that abdominal US exhibits substantial diagnostic accuracy, sensitivity, and specificity in detecting intra-abdominal injuries, with significant positive (PPV) and negative (NPV) values, indicating its reliability in confirming or ruling out intra-abdominal injuries. When restricting the statistical analysis to patients with parenchymal injury without free fluid, the values were as follows: TPs were 24, TNs 8, FPs 15, and FNs 10, with 71% sensitivity, 35% specificity, 62% PPV, 44% NPV, and 56% accuracy [47,48].

Moylan et al. aimed to demonstrate an association between positive FAST and therapeutic laparotomy in normotensive blunt abdominal trauma. The results were comparable to standard CT, with a reported FAST sensitivity of 75.8%, reaching 100% when the patient presented with unstable vital signs and extensive peritoneal fluid collection [48]. Such an association cannot be found in the case of a negative FAST, as this may result in missing patients who still require a laparotomy [48].

A study from Becker et al. was aimed at evaluating the performance of FAST in polytrauma patients, represented by patients having a high Injury Severity Score (ISS), and found that the sensitivity in patients with an ISS > 25 was 65.1%, whereas patients with an ISS < 25 had a sensitivity of 86.4%. This suggests that the FAST exam has a lower accuracy in the context of multiple blunt trauma patients and that patients with a high ISS have an increased risk of having occult US injuries [49].

Thus, the FAST exam has a limited impact in the diagnosis of intraperitoneal parenchymal injuries due to its low sensitivity, although it has a good specificity, comparable to standard CT. Special care should be taken in children, polytrauma patients and patients without free intraperitoneal fluid, as the result of US is not reliable.

2. Pericardiac effusion and tamponade.

A study from Tayal et al. on a sample of 32 patients with anterior chest trauma found that eFAST was 100% sensitive and 100% specific for the detection of pericardial effusion, compared to previous more-invasive procedures, like pericardiocentesis, pericardial window, and thoracotomy [18].

Studies with larger samples have confirmed the reliability of eFAST in emergency pericardial assessment. A 2019 meta-analysis included 75 studies with a total of 24,350 patients and reported a sensitivity of 91% and a specificity of 94% for the detection of pericardial effusion using eFAST [50].

Thus, eFAST can be considered a reliable test for emergency pericardial assessment, with results comparable to CT in terms of both sensitivity and specificity.

3. Pneumothorax.

A study by Kirkpatrick et al. described the superiority of lung US compared to supine AP chest X-ray for the detection of PTX in trauma patients when extending the abdominal US examination to include the lungs [51]. The authors provided insight into the potential of chest sonography as a valuable adjunct to trauma assessment. In this study, lung US demonstrated twice the sensitivity of chest X-ray (48.8% vs. 20.9%), while both modalities demonstrated high specificity (99.6% vs. 98.7%) [3].

Blavias et al. showed similar results. When compared to flat AP chest radiography with CT as the criterion standard, US exhibits higher sensitivity in diagnosing PTX (99.2% vs. 75.5%). In addition, US enables sonologists to distinguish between a small, medium, or large PTX with substantial agreement with CT results [10].

Furthermore, Soldati et al. reported the diagnostic accuracy of US in detecting occult PTX and assessing PTX extension. In their study, 25 of 218 trauma patients had PNX on CT. Of these, only 13 of 25 PTX cases (52%) were detected on chest X-ray (sensitivity, 52%; specificity, 100%), while 23 of 25 PTX cases (92%) were identified on lung US, with one false positive (sensitivity, 92%; specificity, 99.4%). In 20 of 25 cases, there was agreement between the CT lung scan and the lung US scan regarding the extent of the PTX, with a mean difference of 1.9 cm (range, 0 to 4.5 cm) in the localisation of the retroparietal air extension [52].

These data show that lung US performed in the ED is more sensitive and has a higher NPV than AP chest radiography for PTX. It demonstrates almost equivalent accuracy to the reference standard (CT scan) in detecting occult PTX and its extension [52,53,54].

4. Pleural effusion.

Lung US is a highly sensitive and specific exam for the detection of pleural effusion, using CT as the reference criterion.

A study by Lichtenstein et al. aimed to compare lung US and chest radiography in the detection of pleural effusion, along with alveolar consolidation and alveolar-interstitial syndrome, in 32 patients with ARDS. This study found that chest radiography had a sensitivity of 39%, specificity of 89%, and accuracy of 45% in detecting pleural effusion, while lung US had 92% sensitivity, 93% specificity, and 93% accuracy in detecting pleural effusion. The presence of US artefacts, such as comet tails, was used as a negative prognostic marker in detecting pleural effusion [55]. Another study by Akoglu et al. showed even more favourable results, calculating the sensitivity and specificity of lung US to be both 100%, in a study population of 132 patients [56].

Lung US has become an integral part of the daily medical examination in various wards, including the ED. There has been a remarkable growth in publications on lung US over the last decade. In particular, the onset of the COVID-19 pandemic further accelerated the use of lung US due to its ability to provide comprehensive clinical information through bedside examinations that are safe, repeatable, and reliable [57].

5. Comparison with CT and DPL.

Prior to the introduction of US as a reliable diagnostic tool for trauma patients, DPL and CT were the main tests used to assess injury and the presence of fluid in trauma patients.

A study by Akoglu et al. aimed to compare the efficacy of eFAST and CT in a sample of 132 abdominal trauma patients and 130 thoracic examinations. In this study, the AUC of eFAST was 0.71 for abdominal free abdominal fluid, 0.87 for pneumothorax, and 1 for pleural effusion, with an overall sensitivity of 42.9% and specificity of 98.4% [56].

In conclusion, each of these diagnostic modalities has unique advantages and disadvantages. However, DPL remains the most sensitive test for mesenteric and hollow viscus injuries and is less commonly performed due to its invasive nature [58,59,60]. CT scanning is an expensive, reliable test that can detect solid organ injuries and evaluate the retroperitoneum, but its sensitivity and specificity for blunt bowel and mesenteric injuries are not superior to DPL. Furthermore, exposure to ionized radiation limits its use. In the emergency setting, FAST remains a rapid, non-invasive technique that can be repeated several times, including during resuscitation [30,61,62,63,64].

6. US in patients with or without free abdominal fluid.

A major drawback to the accuracy of FAST is its reduced sensitivity in detecting intra-abdominal injuries with minimal or no free peritoneal fluid [28].

A study by Tso et al. on the use of US in the evaluation of patients with BAT showed that the patients with a false positive diagnosis did not have free peritoneal fluid on CT or DPL. Furthermore, when considering only patients with free intraperitoneal fluid, the sensitivity was 91% [32].

Another study by Nnamonu et al. showed similar results regarding the difference in the diagnostic ability of US between abdominal injuries with and without free fluid, showing a decrease in PPV from 94% to 62%; in NPV from 75% to 44%; and in accuracy from 91% to 56% when no free fluid was present [47].

We can, therefore, conclude that eFAST has optimal diagnostic value when applied to BAT patients, but should be considered reliable for the detection of intraparenchymal abdominal injuries (Table 1).

### 3.4. eFAST—Trauma Management in the ED

The eFAST exam can be integrated into the ABCDE primary survey protocol for trauma management. It is valuable in unstable patients undergoing resuscitation because single-view sonographic examinations can assess trauma injury and help diagnose complications found on the ABCDE examination.

The first step is to assess airway patency (A) and check for possible obstruction or misplacement of the oro-tracheal tube, as an intubated patient with suboptimal gas exchange may have a misplaced oro-tracheal tube. eFAST can confirm correct oro-tracheal tube placement by detecting the gliding sign and B-lines in a bilateral chest scan at the P1 and P2 parasternal windows. B-lines, which are absent in PTX, appear statically (comet-tail artifacts) in an oro-tracheal tube malposition. In the case of emergency cricothyroidotomy for glottic or supraglottic obstruction, sonography reduces the time required for the procedure, increases the chances of success by allowing a better anatomical evaluation of the cervical structures, reduces the chances of damaging anomalous vessels, and allows the confirmation of correct needle placement in the trachea, especially in obese patients and those with abnormal neck anatomy [65,66,67].

US can also be used to confirm the correct placement of the endotracheal tube in the trachea or to rule out incorrect placement in the oesophagus [68].

In respiratory assessment (B), US can be used to assess a tachypnoeic or dyspnoeic patient by performing a two-view US of the chest to rule out tension PTX. Bilateral parasternal US of P1 and P2 reduces the time to guide thoracic decompression and thoracentesis [4,69].

According to ATLS, eFAST supports the assessment of circulation (C) in ABCDE to detect hypovolaemia due to haemopericardium, haemothorax, and haemoperitoneum. Free fluid appears as an anechoic band. In the pericardium, it accumulates mainly around the low pressure chambers, most commonly the right atrium and right ventricle [70]. In trauma, hypovolaemia is the most common cause of shock, which is treated with intravenous fluids, except in the case of cardiogenic shock. In this setting, sonography can assess the circulatory status (hyperkinetic heart syndrome or collapsed inferior vena cava) and assist with difficult venous access placement [4,68,69].

The role of eFASt in cardiopulmonary resuscitation (CPR) remains an area of growing interest and ongoing debate. While traditional ATLS protocols rely heavily on pharmacologic interventions, such as high-dose adrenaline, recent evidence has raised concerns about the potential for increased mortality, particularly in cases where the underlying reversible causes of cardiac arrest are not promptly identified and treated. Recent studies highlight the importance of early aetiological diagnosis and suggest that targeted intervention, enabled by tools such as eFAST, may improve outcomes [71]. 

During cardiopulmonary resuscitation, it is recommended to minimize any interruptions in resuscitation efforts and to identify and treat potentially reversible causes. Therefore, the rapid and appropriate use of US in emergencies could improve the outcome of these patients. In cases of pseudo-pulseless electrical activity (PEA), characterized by the absence of effective contractile activity, the use of US has revealed some contractile activity, known as wall motion, suggesting the potential for a subclinical return to spontaneous circulation. US allows the diagnosis of underlying causes of cardiorespiratory arrest, such as cardiac tamponade or tension pneumothorax [72].

The fourth step of the ABCDE assesses disability (D), including consciousness and neurological status. Authors have suggested that the eFAST examination could check for signs of intracranial hypertension: as the optic nerve is the only cranial nerve surrounded by a myelin sheath, a change in its transverse diameter (more than 6 mm) has been suggested as a potential indicator of intracranial hypertension. Such structures should be visualised by directing the ultrasound beam perpendicularly to the diameter of the optic nerve, measured at 3 mm from the globe. However, it is not included in the ED assessment of trauma patients [4,73,74].

The fifth step of the ABCDE survey involves a comprehensive patient’s examination to identify any additional injuries once acute emergencies have been ruled out. Further evaluation may involve the use of US for various purposes, such as the identification of fractures (sternum and ribs in BAT); the monitoring of pulmonary contusions [75]; the evaluation of pneumothorax following decompression or thoracocentesis or the detection of occult pneumothorax; the assessment of abdominal parenchymal lesions for potential internal haemorrhage (e.g., hepatic, splenic, or renal contusions and haematomas); the assessment of soft tissues for haematomas, subcutaneous emphysema, ocular, muscular, and tendinous lesions; and the management of local anaesthesia [7,76].

As we mentioned above, US is an integral part of trauma resuscitation. Damage Control Resuscitation (DCR) is a strategy for managing trauma patients by rapidly treating bleeding and coagulopathy while preventing hyperperfusion-related damage. It includes hypovolaemic resuscitation, early coagulation support (ECS), and the prevention of hypothermia.

Hypovolaemic resuscitation should restore different blood pressures (BP) depending on the severity of the trauma.

1. Systolic BP 70 mmHg, for penetrating trauma;
2. Systolic BP 90 mmHg, for blunt trauma;
3. Systolic BP 110 mmHg, for head trauma [77,78].

The early coagulation support protocol (ECS) must be overseen by the team leader, who monitors fluids and blood in unstable patients based on one major criteria (the presence of uncontrollable bleeding) and at least one of five minor factors (systolic AP < 100 mmHg; lactates > 5.0 mmol or 45 mg/dL; BE < −6; Hb < 9 g/dL; INR > 1.5) [79].

In this setting, a standardized diagnostic algorithm is essential for the management of haemodynamically unstable patients in the ED. While providing fluid resuscitation and coagulation support, the team leader decides with the surgical team whether the patient should go to the operating room (OR). If the OR is not required, the patient can be transferred to the Intensive Care Unit (ICU) or an appropriate ward. If surgery is needed, a CT scan, X-ray, or angiography may follow. REBOA (Resuscitative Endovascular Balloon Occlusion of the Aorta) placement may be considered in the OR. After surgery, further imaging will be used to assess the patient’s condition before transfer to the ICU [80,81].

Sonography can be incorporated at several points in such a diagnostic algorithm, guiding clinical decisions and quickly directing the patient to the most appropriate treatment. As mentioned above, it is highly effective in ruling out traumatic chest conditions such as PTX, pericardial effusion, and haemothorax [3,82] as the lung-sliding sign is an excellent negative predictor [83,84,85]. In addition, eFAST can help to rule out pulmonary embolism in patients who cannot undergo a CT scan or who are unstable by detecting indirect signs, such as the right heart chamber’s enlargement.

FAST can be used to rapidly assess patients with blunt abdominal trauma during resuscitation. Following a positive FAST examination in a haemodynamically stable patient, CT is recommended to confirm possible organ injury, whereas its use in haemodynamically unstable patients may help to localise the potential site of bleeding. The identification of fluid with characteristics suggestive of blood reduces diagnostic time and helps to guide the patient directly to laparotomy without further diagnostic procedures, such as peritoneal lavage or abdominal CT [86]. A negative eFAST can reliably rule out the presence of intraperitoneal fluid, limiting the need for further investigation.

### 3.5. eFAST—Limitations

Despite its many advantages, eFAST has some limitations.

It may not always detect small amounts of blood or injuries in certain areas. It is neither sensitive nor specific for detecting solid organ injury without intraperitoneal fluid [32]. It has limited reliability in blunt abdominal trauma (BAT) due to a significant decrease in accuracy for detecting intraparenchymal abdominal injuries that do not produce free fluid [47]. In addition, the retroperitoneal area has a limited sonographic window, making CT or MRI more reliable, especially in obese patients, where perirenal fat may mimic free fluid [87]. A possible solution to the low sensitivity of eFAST in detecting BAT may be serial testing, as the amount of blood is likely to increase over time in patients with active intraperitoneal haemorrhage [1]. High clinical suspicion or worsening conditions may suggest repeat testing at short intervals [7,88,89].

Another limitation of the FAST exam in paediatric patients is its reduced sensitivity (80%) due to the lower amount of detectable free fluid, particularly in the detection of haematoperitoneum [90]. In addition, many studies exclude this group, despite the potential to reduce CT-related radiation exposure.

The FAST exam also has limited reliability in polytrauma patients [49]. The detection of free fluid does not always indicate the presence of blood. The presence of ascites, ovarian cyst rupture, or peritoneal dialysis may cause false positives, requiring careful assessment and clinical correlation. Due to the changing echogenicity of blood, which is similar to that of soft tissue, multi-window scanning may be useful, especially over the lower slopes of organs where blood tends to pool.

It may also be less effective in patients with certain comorbidities that interfere with US visualisation. US is limited in patients with comorbidities such as severe obesity or subcutaneous emphysema, due to the air shadowing of underlying structures on US, or chronic lung disease, which may reduce the accuracy of cardiac images due to lung hyperinflation. In these patients, eFAST findings should be integrated with other imaging modalities.

The quality and accuracy of the examination is highly dependent on the experience and expertise of the operator. Operator dependency remains a challenge, highlighting the importance of structured training and competency assessment to improve accuracy. Many physicians lack the necessary training to perform and to interpret such an examination correctly. In a review by Jang et al., 24% of physicians performing their first 10 exams had multiple uninterpretable views or misinterpreted images, and a single uninterpretable view, along with poor gain, suboptimal depth, and backward image orientation, occurred in 48% of the same sample, leading to a wide range of common technical mistakes that could compromise the reliability of eFAST [91,92]. For this reason, a specific didactic and hands-on training programme must be arranged. Performing a certain number of eFAST examinations, ranging from 20 to 50, seems to result in adequate diagnostic competence in clinicians [6,15].

## 4. Discussion

eFAST has changed the landscape of trauma assessment due to its speed, non-invasiveness, and effectiveness in detecting free fluid, pneumothorax, and pericardial effusion. Ultrasound offers several advantages, including real-time imaging, repeatability, and the avoidance of radiation exposure. However, despite these advantages, challenges remain in optimizing its accuracy. Although eFAST has been shown to be valuable, certain limitations remain with regard to its sensitivity in detecting injuries without free fluid or with small-volume haemorrhages. The reliability of eFAST in polytrauma patients is also debated, as non-haemorrhagic fluid collections may lead to false positives. To determine whether the anaechoic area is a vessel or a laceration, performing a colour Doppler scan over the area of interest may be helpful. Very large and actively bleeding lacerations may show a small amount of colour flow, but the appearance of the colour flow will be markedly different from that seen over the normal adjacent veins and arteries.

In addition, contrast-enhanced ultrasound (CEUS) can also be a useful method that has recently emerged in emergency situations. Its main characteristic compared to other ultrasound techniques is that it does not have extra vascular phases. The use of contrast in traumatised patients with hypovolaemic shock who cannot have a CT scan is very advantageous. It is useful as a contrast agent in patients who are allergic to CT contrast and for monitoring traumatic lesions for which conservative treatment has been chosen, allowing the number of follow-up CT scans to be reduced [3,4,93,94].

The advantage of eFAST is that it can be performed at the pre-clinical stage in emergencies and in ambulances. It may also be of interest for use in pre-hospital and austere settings, where rapid decision making is critical and CT scans are not available [95].

The role of US in CPR is controversial. Studies suggest that it can identify the causes of cardiac arrest, but others argue that it can prolong the assessment and interfere with compressions. Guidelines should focus on optimizing its use in CPR.

Operator dependency remains a major limitation of eFAST, highlighting the need for structured US training programmes. Dedicated courses, including training in remote imaging, could improve recognition skills and improve diagnostic accuracy. New research may further support the idea that trained nurses who can perform eFAST can provide quality support to triage activities, enabling the activation of the best care pathway for the patient, with a focus on safety and care efficiency [96]. The burden of artificial intelligence over the last decade may further revolutionise this field by providing automated image interpretation to assist less-experienced clinicians [97].

## 5. Conclusions

The implementation of eFAST in the diagnostic work-up of abdominal and thoracic trauma may be beneficial for the overall outcome of trauma patients, as ultrasound has better specificity than X-ray and a comparable NPV to CT and DPL. Thus, the integration of bedside US into daily emergency department practice may reduce the risk of radiation exposure, the need for patient transport, hospital costs, and redirect patient management [33,57]. A positive eFAST exam for BAT still requires CT for confirmation and the better localisation of damage, as sonography has no reliable sensitivity for abdominal injuries, but gives reliable results with high PPV values for thoracic trauma, such as PTX, pericardial effusion, and cardiac tamponade. The eFAST exam has great potential to be integrated into the ABCDE primary survey, providing a rapid tool for the assessment of organ failure, lesions, and the management of damage-control resuscitation. eFAST provides pre-, intra-, and post-operative guidance, assists with invasive procedures, monitors treatment efficacy, and detects complications, thanks to its ability to be performed in series. Efforts should be made to provide specific eFAST training for emergency physicians and surgeons to improve the use of sonography in trauma management.

## Figures and Tables

**Table 1:** Comparison of strengths and limitations of eFast.

Condition	Strengths	Limitations
**Blunt Abdominal Trauma (BAT)**	- High sensitivity for detecting haemoperitoneum and free fluid. - Non-invasive. - Reliable in detecting traumatic injuries with fluid presence.	- Lower sensitivity for detecting visceral parenchymal injury without free fluid. - Accuracy reduced in polytrauma patients with high ISS.
**Pericardiac effusion**	- Highly accurate for detecting pericardial effusion. - Comparable to invasive methods in chest trauma cases.	- Limited use for other pericardial or cardiac conditions. - Might not detect very small or loculated effusions.
**Pneumothorax**	- Higher sensitivity for PTX detection than chest X-ray. - Helps identify size and location of PTX.	- Requires skillful use and may miss small PTXs in certain cases. - Does not replace CT in cases of extensive PTX.
**Pleural Effusion**	- High sensitivity and specificity for pleural effusion. - More accurate than chest X-ray.	- May not detect small effusions or complex effusions effectively.

## Data Availability

Not applicable.

## Abbreviations

ABCDE	Airways, Breathing, Circulation, Disability, Exposition
AP	Anteroposterior
ARDS	Acute Respiratory Distress Syndrome
ATLS	Advanced Trauma and Life Support
AUC	Area Under Curve
BAT	blunt abdominal trauma
BP	blood pressure
CT	Computer Tomography
DCR	Damage Control Resuscitation
DPL	diagnostic peritoneal lavage
ECG	Electrocardiography
ECS	early coagulation support
ED	emergency department
eFAST	Extended Focused Assessment with Sonography for Trauma
FAST	Focused Assessment with Sonography for Trauma
FN	false negative
FP	false positive
GHz	Giga-Hertz
ICU	Intensive Care Unit
ISS	Injury Severity Score
IV	Intravascular
LUQ	Left upper quadrant
MRI	Magnetic Resonance Imaging
NPV	Negative Predictive Value
OR	operating room
PPV	Positive Predictive Value
PTX	pneumothorax
REBOA	Resuscitative Endovascular Balloon Occlusion of the Aorta
RUSH	Rapid Ultrasound in Shock and Hypotension
TN	true negative
TNR	True Negative Rate
TP	true positive
US	ultrasound
